# Investigating the Correlation Between Clostridioides difficile Infection and Vitamin D Deficiency

**DOI:** 10.7759/cureus.39970

**Published:** 2023-06-05

**Authors:** Ayham Khrais, Anna G Mathew, Aaron Kahlam, Alexander Le, Anmol Mittal, Siddharth Verma

**Affiliations:** 1 Department of Medicine, Rutgers University New Jersey Medical School, Newark, USA; 2 Department of Gastroenterology and Hepatology, East Orange Veteran's Affairs Medical Center, East Orange, USA

**Keywords:** vitamin d deficiency, c. difficile, clostridioides difficile infection, food and nutrition, vitamin d level

## Abstract

Introduction: *Clostridioides difficile* infection (CDI) is the most common healthcare-associated infection in the US. Symptoms include watery diarrhea, nausea, and anorexia and it can present with leukocytosis on laboratory evaluation. Treatment is based on disease severity and recurrence. Despite antibiotic usage being the highest risk factor for infection, they are also the first-line treatment for initial CDI. Prevention of CDI mostly involves good hand hygiene, antibiotic stewardship, and appropriate precautions when interacting with infected individuals. Vitamin D deficiency (VDD) has been linked to CDI, however, there is limited insight into the correlation between both states. Our aim was to further investigate the potential link between VDD and CDI.

Methods: Data were obtained from the National Inpatient Sample (NIS) from 2016 to 2019. Patients with CDI were identified and stratified based on a diagnosis of VDD. Primary outcomes were mortality, CDI recurrence, ileus, toxic megacolon, perforation, and colectomy. Chi-squared and independent t-tests were performed to assess categorical and continuous data, respectively. Multiple logistic regression was used to control for confounders.

Results: Patients with VDD had higher rates of CDI recurrence (17.4% versus 14.7%, p<0.05), but lower rates of mortality (3.1% versus 6.1%, p<0.05). Differences in rates of ileus, toxic megacolon, perforation, and colectomy were statistically insignificant. Length of stay was higher in the VDD group (10.38 days versus 9.83 days). Total charges were lower in the VDD group ($93,935.85 versus $102,527.9).

Discussion: CDI patients with comorbid VDD are at higher risk for the recurrence of CDI. This is likely due to the role of vitamin D in the expression of intestinal epithelial antimicrobial peptides, macrophage activation, and maintenance of tight junctions between gut epithelial cells. Furthermore, vitamin D plays a role in maintaining a healthy gut microbiome. Alternatively, deficiency results in poor gut health and detrimental changes to the gut microbiome. In effect, VDD promotes the proliferation of *C. difficile* within the large colon, resulting in an increased predisposition for CDI.

## Introduction

*Clostridioides difficile* infection (CDI) is the most common healthcare-associated infection in the US, accounting for 15% of all such infections [1]. Characterized by inflammation and damage to the colonic mucosa from toxins produced by *C. difficile* colonization, the infection is acquired by the fecal-oral route, especially more likely once the colonic mucosa has been impaired by antibiotic use [2]. The infection often presents as watery diarrhea (≥3 unformed stools/24 hours), nausea, anorexia, and leukocytosis with a neutrophilic predominance [3]. Diagnosis of CDI is primarily based on the presence of the aforementioned symptoms, coupled with the identification of *C. difficile* organisms/toxins in stool [3]. CDI treatment is based on disease severity. Despite antibiotics usage being the highest risk factor for infection, they are also the first-line treatment for initial CDI, specifically vancomycin or fidaxomicin [4]. Recurrent infection often follows antibiotic treatment, leading to the use of other approaches, such as fecal transplant or oral administration of nontoxigenic *C. difficile* spores [5]. As per recent estimates by the Centers for Disease Control, *C. difficile* infects approximately 500k Americans per year, with an annual US economic burden estimated to be $796 million [6]. Additionally, CDI is associated with substantial morbidity and mortality in the general population, as well as in specific at-risk groups (e.g. elderly, hospitalized patients, immunocompromised) [6]. As we anticipate an increasingly aging population, the incidence of CDI is expected to continue to grow in the coming years [6].

Additionally, CDI has been previously associated with several nutritional deficiencies, including vitamin D deficiency (VDD) [7,8]. VDD affects approximately one billion people across the world, with previous literature demonstrating high prevalence in both developing and developed countries [9]. Causes of VDD can range from decreased sun exposure/dietary intake to diminished endogenous synthesis or increased hepatic catabolism [10]. VDD can present asymptomatically or as nonspecific fatigue/muscle ache in mild cases; however, more severe cases can present with rickets or osteomalacia [11]. Diagnosis of deficiency is based on serum levels of 25-hydroxyvitamin D. Management of VDD begins with oral supplementation for eight weeks with 6000 IU daily, with considerations of increasing dosage or augmenting with calcitriol in more severe cases [12].

Although CDI has been previously associated with VDD, there has been limited insight into the direct impact of this nutritional deficiency on the morbidity and morbidity, and mortality of hospitalized CDI patients [7,8]. This study investigates the clinical outcomes of patients hospitalized for CDI with a history of VDD.

## Materials and methods

Data source

Data were extracted from the National Inpatient Sample (NIS), the largest public all-payer inpatient database containing information on more than seven million hospital stays in the United States. The NIS was developed by the Agency for Healthcare Research and Quality, and contains no patient or hospital identifiers, providing a nationally representative set of data representing 20% of all discharges from hospitals within the U.S. Sample weight is applied annually, enabling precise estimates. In this study, in-hospital cases among the NIS data were compiled for patients hospitalized from 2016 to 2019 using the International Classification of Diseases, Tenth Revision, and Clinical Modification (ICD-10 CM) codes. Informed consent was not necessary for this project as data were obtained from a publicly-available database.

Study design

This is a cross-sectional study. Inclusion criteria consisted of patients 18 years old or older hospitalized in the U.S. and diagnosed with CDI between 2016 and 2019. Patients were stratified based on the presence of ICD-10 codes for VDD. Primary measured outcomes included inpatient mortality, length of stay, hospital costs, and complications of CDI including recurrent infection, ileus, toxic megacolon, perforation, and colectomy. Demographic information such as age, sex at birth, and race were analyzed as well.

Statistical analysis

The Statistical Product and Service Solutions (SPSS) Statistics 24 (IBM Corp., Armonk, NY, USA) software was used to conduct statistical analyses. Independent t-tests and Chi-squared were used to analyze continuous and categorical data, respectively. Multiple logistic regression was used to analyze outcomes while controlling for confounding variables, including age, sex at birth, and race. Statistical significance was characterized by a p-value <0.05. Adjusted odds ratios (AOR) and associated 95% confidence intervals (CI) were calculated. Tables containing obtained data were generated using a combination of Microsoft Excel and Microsoft Word.

## Results

From 2016 to 2019, 73,745 individuals were admitted with CDI, of whom 1,756 were diagnosed with VDD. On average, patients with and without VDD were of similar ages (66.12 years versus 66.26 years, respectively) (Table 1). The majority of both groups were female, however, the VDD group comprised a greater percentage of females than the non-VDD group (63% versus 57.3%, respectively). In terms of racial composition (Table 1), both VDD and non-VDD groups were mostly Caucasian (71.9% versus 74%), followed by Black (16% versus 13%) and Hispanic (7.2% versus 8.3%).

**Table 1:** Patient Demographics n = sample size, SD = standard deviation

		No Vitamin D Deficiency (n=71,989)	Vitamin D Deficiency (n=1,756)
Mean Age in Years (SD)		66.26 (16.632)	66.12 (16.967)
Sex	Male (%)	30,743 (42.7)	650 (37)
	Female (%)	41,241 (57.3)	1,106 (63)
Race	Caucasian (%)	51,905 (74)	1,238 (71.9)
	Black (%)	9,134 (13)	275 (16)
	Hispanic (%)	5,825 (8.3)	124 (7.2)
	Asian/Pacific Islander (%)	1,175 (1.7)	35 (2.1)
	Native American (%)	510 (0.7)	10 (0.6)
	Other (%)	1,622 (2.3)	38 (2.2)

In terms of outcomes (Table 2), patients with VDD had higher rates of recurrent CDI (17.4% versus 14.7%) and longer lengths of stay (10.38 days versus 9.83 days). Those without VDD had higher rates of mortality (6.1% versus 3.1%), ileus (3.6%), and costlier total hospital charges ($102,527.9 versus $93,935.85). No significant difference in the rates of toxic megacolon, colonic perforation, and colectomy was observed between both groups. Differences in mortality, CDI recurrence, and ileus were statistically significant (Table 3).

**Table 2:** Primary and Secondary Outcomes n = sample size, CI = confidence interval, SD = standard deviation, CDI = *Clostridioides difficile* infection

	No Vitamin D Deficiency (n=71,989)	Vitamin D Deficiency (n=1,756)	Odds Ratio (CI)	p-value
Mortality (%)	4,385 (6.1)	54 (3.1)	0.489 (0.372 to 0.643)	<0.001
Recurrent CDI (%)	10,611 (14.7)	305 (17.4)	1.216 (1.073 to 1.378)	0.002
Ileus (%)	2,568 (3.6)	45 (2.6)	0.711 (0.527 to 0.958)	0.024
Toxic Megacolon (%)	59 (0.1)	2 (0.1)	1.39 (0.339 to 5.694)	0.646
Perforation (%)	360 (0.5)	7 (0.4)	0.796 (0.376 to 1.685)	0.551
Colectomy (%)	4,855 (6.7)	109 (6.2)	0.915 (0.752 to 1.113)	0.375
			Standard Error (CI)	p-value
Mean Length of Stay (SD)	9.83 days (12.773)	10.38 days (11.909)	0.308 (-1.145 to 0.063)	0.039
Mean Total Charges (SD)	$102,527.9 (204,072.6)	$93,935.85 (162,973.7)	4,913.296 (-1,038.02 to 18,222.06)	0.04

Upon adjustment for confounding variables, including age, sex at birth, and ethnicity (Table 3), differences in mortality and recurrent CDI were statistically significant.

**Table 3:** Multiple Logistic Regression n = sample size, ACI = adjusted confidence interval, SD = standard deviation, CDI = *Clostridioides difficile* infection

	Adjusted Odds Ratio (ACI)	Adjusted p-value
Mortality (%)	0.499 (038 to 0.656)	<0.001
Recurrent CDI (%)	1.201 (1.058 to 1.362)	0.005
Ileus (%)	0.777 (0.573 to 1.052)	0.103
Toxic Megacolon (%)	1.958 (0.475 to 8.083)	0.353
Perforation (%)	1.01 (0.475 to 2.145)	0.979
Colectomy (%)	0.844 (0.69 to 1.032)	0.099

## Discussion

The results of our study show an association between VDD and rates of recurrent CDI in hospitalized patients. There are several proposed mechanisms by which this deficiency leads to worsened clinical outcomes for CDI patients.

Previous literature has investigated the role of vitamin D in normal intestinal homeostasis and barrier function. Firstly, vitamin D increases the expression of several antimicrobial peptides in the intestinal epithelium, such as cathelicidins (LL-37) and β-defensin, which serve critical roles in maintaining gut microbiome integrity [13,14]. Specifically, vitamin D receptors activate macrophages to upregulate the expression of cathelicidin, an endogenous peptide with broad-spectrum antimicrobial activity at natural barrier sites, such as the gut lining [13]. This peptide is expressed by gut epithelial cells and serves a significant role in the first line of defense [13]. Vitamin D has also previously been shown to serve a protective effect on maintaining the structural integrity of the intestinal lining [15]. Specifically, activation of the vitamin D receptor increases the expression of several intracellular junction proteins that compose the tight junctions between epithelial cells of the gut [15]. Thus, patients with VDD may experience impaired immune function through decreased expression of antimicrobial peptides and compromised epithelial barrier integrity, contributing to their increased odds of developing recurrent CDI.

Additionally, vitamin D serves a significant role in protecting the gut microbiome. In fact, vitamin D supplementation in deficient patients significantly improved gut microbiota diversity [16]. Specifically, supplementation leads to an increase in the *Bacteroidetes *to *Firmicutes *ratio (typically lowered in CDI patients), as well as an increase in the relative abundance of *Bifidobacterium*, which has been previously shown to inhibit the growth of *C. diff* and prevent progression to infection [16,17]. Accordingly, patients with VDD may be at greater risk of impaired species richness in their gut microbiome. Differences in microbiota across patients with recurrent CDI have not been fully elucidated; however, previous literature has established the association between a loss of bacterial diversity and the likelihood of recurrent CDI [18]. Thus, patients with VDD may be at additional risk of recurrent CDI due to their compromised gut microbial diversity.

Alongside increased odds of recurrent CDI, our study also found an inverse association between VDD and mortality of hospitalized CDI patients. A majority of prior studies have shown that vitamin D levels are inversely correlated with mortality [19,20]. However, our results directly contradict these previous findings. Others have found a U-shaped correlation between VDD and general mortality, with mortality being high in patients with very low and very high vitamin D levels [20]. This could be due to sicker patients being advised to take vitamin D supplementation in an attempt to improve their overall mortality, providing a fictitious inverse correlation between VDD and mortality [20]. We hypothesize that this discrepancy may be attributed to this phenomenon.

There are a few important limitations of this study. Firstly, this project was based on the NIS database, which is entirely reliant on the accuracy and precision of billing codes inputted by healthcare providers. Inconsistent usage of this information can misrepresent the prevalence of VDD in patients hospitalized for CDI. Specifically, in more severe or acute cases, vitamin D levels may not be recorded, so they could not be included in our analysis. This may bias our data toward underestimating the prevalence of VDD or misunderstanding its impact on clinical outcomes in acute cases. Additionally, our study was unable to stratify patients by severity of VDD, precluding more advanced analysis of the data and limiting the conclusions drawn from our results. Finally, the NIS database considers each encounter as a case, rather than individual patients, thus possibly confounding the outcomes due to repeat admissions. Despite these limitations, this study was able to evaluate patient demographics and clinical outcomes on a national scale over the span of three years. Additionally, the use of a multivariate logistic regression analysis allowed us to evaluate risk factors associated with worsened CDI outcomes after adjustment for several confounding demographic factors and comorbidities.

## Conclusions

In summary, patients hospitalized for infection by *C. difficile* with concomitant VDD may be at an increased odds of developing recurrent CDI. These findings may be used to guide the clinical management of CDI, specifically emphasizing that vitamin D supplementation may be considered for hospitalized CDI patients to minimize the chances of reinfection. The results of this study will likely become increasingly relevant with the growing prevalence of hospitalizations for CDI. Further investigation is needed to evaluate the impact of varying degrees of VDD on the chances of developing recurrent CDI. In addition, future research may evaluate the impact of vitamin D supplementation even in non-deficient patients in improving CDI outcomes.
